# Comparative Effects of O_2_ Plasma and Thermal Annealing on the Physicochemical and Electrical Characteristics of iCVD pV3D3 Dielectric Films

**DOI:** 10.3390/polym18212700

**Published:** 2026-10-31

**Authors:** Dong Yun Sung, Ji Yun Lee, Seo Yeon Choi, Hyung Jun Kim, Sun Jae Kim, Min Ju Kim, Sarah Eunkyung Kim, Jong Kyung Park, Seul Ki Hong

**Affiliations:** 1 Department of Semiconductor Engineering, Seoul National University of Science & Technology, Seoul 01811, Republic of Korea; dysung@seoultech.ac.kr (D.Y.S.); lovving@seoultech.ac.kr (J.Y.L.); tjdus0642@seoultech.ac.kr (S.Y.C.); eunkyung@seoultech.ac.kr (S.E.K.); jkpark1@seoultech.ac.kr (J.K.P.); 2 School of Electronics and Electrical Engineering, Dankook University, Yongin 16890, Republic of Korea; hjkimbs@dankook.ac.kr (H.J.K.); minju9062@dankook.ac.kr (M.J.K.); 3 Centre for Materials Science and Nanotechnology, Department of Physics, University of Oslo, 1048 Blindern, NO-0316 Oslo, Norway; sunjae.kim@smn.uio.no

**Keywords:** pV3D3, polymer dielectric, semiconductor processing, O_2_ plasma treatment, thermal annealing, XPS, TLM, C–V

## Abstract

Functional polymers are increasingly considered for semiconductor integration, but their properties may be altered by plasma and thermal processing. Here, 50 nm thick iCVD-grown pV3D3 dielectric films were subjected to O_2_ plasma treatment and thermal annealing at 250 and 300 °C, and the resulting physicochemical and electrical changes were comparatively investigated. O_2_ plasma treatment generated C–O and O–C=O surface bonding states and shifted the Si 2p spectrum toward higher binding energy, while the RMS roughness remained below 0.5 nm. In contrast, thermal annealing caused comparatively limited surface-chemical changes but substantially altered the electrical response. In particular, the 300 °C-annealed sample exhibited strongly suppressed capacitance and a pronounced deviation from the linear resistance–length relationship observed for the reference and lower-temperature conditions. These results indicate that plasma treatment predominantly modifies the near-surface chemistry of pV3D3, whereas elevated thermal processing can induce electrical changes that are not fully captured by surface-sensitive characterization. The combined analysis highlights the importance of thermal-budget control when integrating pV3D3 into semiconductor process flows.

## 1. Introduction

Recent advances in semiconductor packaging and heterogeneous integration have increased the demand for integrating materials with diverse functionalities into semiconductor systems. In this context, functional polymers have attracted considerable attention owing to their tunable physical, chemical, and electrical properties, which offer potential for various semiconductor applications. However, polymeric materials can undergo changes in their chemical structures and physical properties under semiconductor processing conditions, such as thermal treatment and plasma processing, which may subsequently affect their electrical characteristics [1]. Therefore, understanding the effects of processing conditions on the properties of functional polymers is important for evaluating their process compatibility and enabling their integration into semiconductor systems [2,3,4].

Among various functional polymers, poly(1,3,5-trivinyl-1,3,5-trimethylcyclotrisiloxane) (pV3D3) is a cross-linked polymer that can be deposited by initiated chemical vapor deposition (iCVD) [5,6,7,8]. Owing to its dielectric properties and thin-film deposition capability, pV3D3 has been investigated for electronic device applications [9,10,11,12,13]. Studies have also investigated the thermal properties of pV3D3 and its response to plasma treatment in relation to semiconductor processing [14,15].

Previous studies have demonstrated that O_2_ plasma treatment can modify the surface chemistry of pV3D3 and improve its interfacial properties, while thermal treatment can induce structural rearrangement of the cyclosiloxane network [14,15,16]. However, most previous studies have focused either on surface modification, structural evolution, or device implementation separately. A direct comparison of plasma- and thermally induced changes in pV3D3 using both physicochemical characterization and electrical test structures fabricated through a common semiconductor process flow remains limited. In this study, we comparatively investigated the response of 50-nm-thick iCVD-grown pV3D3 films to O_2_ plasma treatment and thermal annealing at 250 and 300 °C. Surface morphology and chemical states were evaluated by AFM and XPS, while the corresponding electrical responses were examined using C–V measurements and resistance–length test structures. By comparing surface-sensitive characterization with electrical measurements obtained after identical downstream fabrication processes, this study aims to identify process-induced changes relevant to the integration of pV3D3 into semiconductor fabrication.

## 2. Materials and Methods

### 2.1. Sample Preparation and Fabrication

A 50 nm thick pV3D3 film was deposited on a 4-inch p-type Si wafer using initiated chemical vapor deposition (iCVD ISAC Research Co, Ltd, Daejeon, Korea). V3D3 (purity 96%, Jihyun Chemical, Hwaseong, Korea) and di-tert-butyl peroxide (TBPO, purity 98%, Aldrich, St. Louis, MO, USA) were used as the monomer and initiator, respectively, with flow rates of 1 and 0.5 sccm, respectively. The V3D3 source, filament, and substrate temperatures were maintained at 60, 200, and 40 °C, respectively. The chamber pressure was maintained at 300 mTorr, and the deposition was performed for 2 h. Following deposition, the samples were divided into four groups according to the processing condition: an as-deposited reference, an O_2_ plasma-treated sample, and samples thermally annealed at 250 and 300 °C. Thermal annealing was performed at 250 and 300 °C for 3 h under vacuum conditions. The annealing temperatures of 250 and 300 °C were selected based on the processing conditions investigated in our previous metal–polymer bonding studies to evaluate the effects of thermal exposure on pV3D3 films under bonding-relevant conditions. O_2_ plasma treatment was performed at 200 W with an O_2_ flow rate of 180 sccm for 60 s. Both treatments were performed on the pV3D3 films after iCVD deposition and prior to photolithography and electrode fabrication.

**Figure 1:**
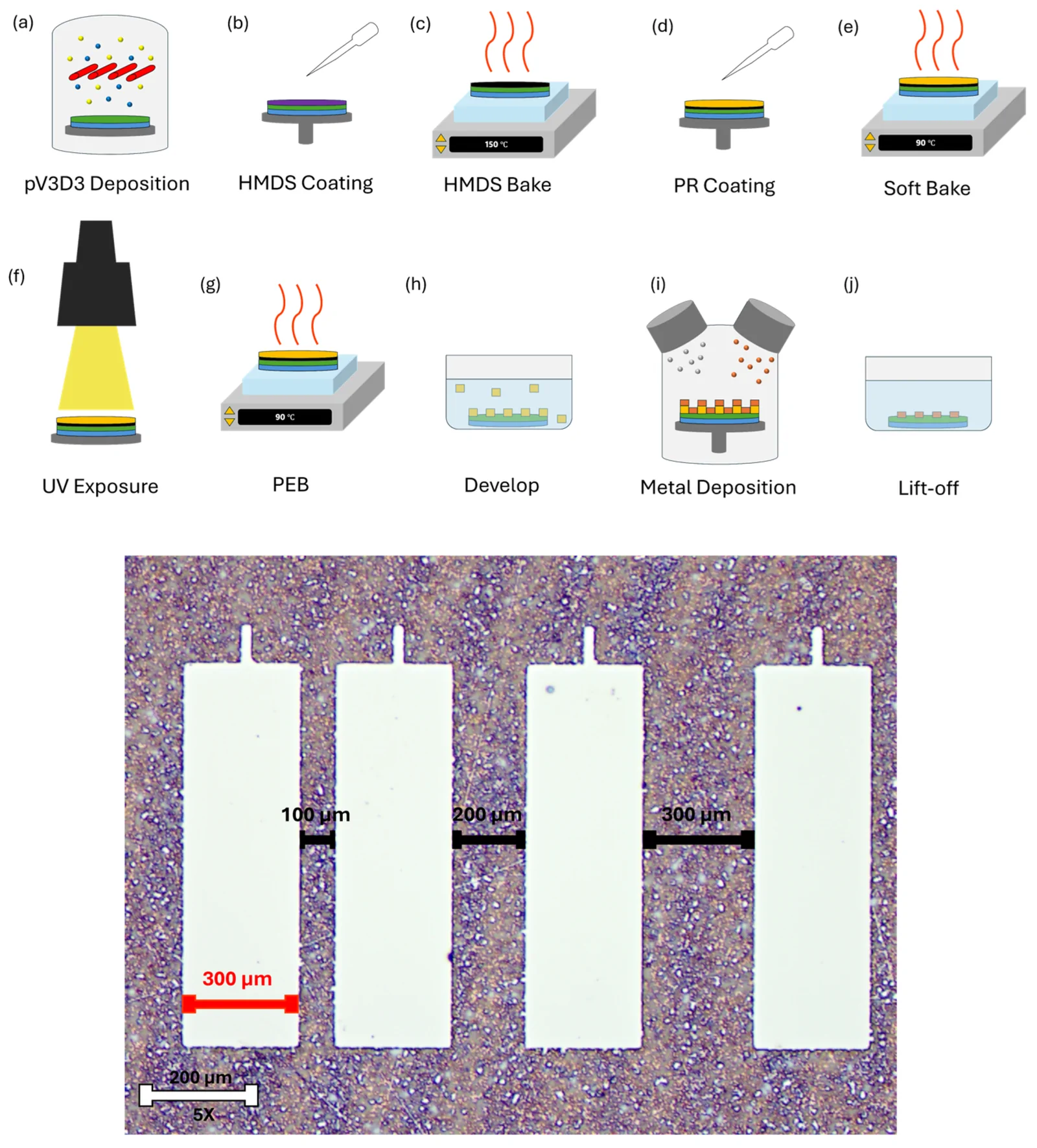
Schematic illustration of the fabrication process for pV3D3 electrical test structures and an optical microscopy image of the fabricated TLM electrodes. Prior to photoresist coating, (**a**) the pV3D3 films were subjected to O_2_ plasma treatment or thermal annealing at 250 and 300 °C, while the reference samples were used as deposited. (**b**,**c**) The HMDS bake was performed at 150 °C, (**d**–**f**) while the (**g**) soft bake and (**h**) post-exposure bake were performed at 90 °C. (**h**–**j**) The TLM structures were fabricated with electrode dimensions of 300 µm × 1000 µm and channel lengths ranging from 100 to 1000 µm. Abbreviations: HMDS, hexamethyldisilazane; PEB, post-exposure bake.

Electrical test structures were subsequently fabricated using photolithography and metallization processes, as schematically illustrated in Figure 1. Following hexamethyldisilazane (HMDS, 95–100 wt%) treatment and photoresist coating, electrode patterns were defined by UV lithography and development. Ti/Cu/Ti layers with thicknesses of 50/300/50 nm, respectively, were then deposited, followed by a lift-off process to form the electrodes. The fabricated structures were used for capacitance–voltage (C–V) and transfer length method (TLM) measurements.

### 2.2. Material Characterization

The physicochemical properties of the pV3D3 films were characterized using X-ray photoelectron spectroscopy (XPS), Fourier-transform infrared spectroscopy (FT-IR), and atomic force microscopy (AFM). XPS was employed to investigate changes in the surface chemical composition and bonding states of pV3D3 induced by O_2_ plasma treatment and thermal annealing. Attenuated total reflectance Fourier-transform infrared (ATR-FTIR) spectroscopy was used to characterize the chemical structure of the as-deposited pV3D3 film. The surface morphology and root-mean-square (RMS) roughness of the reference and O_2_ plasma-treated pV3D3 films were evaluated using AFM to examine whether direct plasma exposure induced nanoscale surface roughening.

### 2.3. Electrical Characterization

The electrical characteristics of the pV3D3 films under different processing conditions were evaluated using capacitance–voltage (C–V) and transfer length method (TLM) measurements. C–V measurements were performed at room temperature at a frequency of 10 kHz using metal/pV3D3/Si structures. The voltage terminal was connected to the top Ti/Cu/Ti electrode, while the ground terminal was connected directly to the Si substrate through a region where the pV3D3 film had been removed. The bias voltage was swept from −6 V to +4 V with a voltage step of 0.5 V. The measured capacitance was normalized by the electrode area (300,000 µm^2^) and expressed as capacitance density to compare the dielectric characteristics of the samples under different processing conditions.

TLM structures with different channel lengths were fabricated to evaluate both the length-dependent conduction through the pV3D3-based structure and the length-independent resistance contribution associated with the metal contacts. The measurements were performed at room temperature by sweeping the applied voltage from −10 V to +10 V with a voltage step of 0.5 V.

The total resistance was analyzed according to

$$R_{\mathrm{total}}=R_{s}\frac{L}{W}+{2R}_{C}$$

where *R_s_* is the sheet resistance, *L* is the channel length, *W* is the channel width, and *R_c_* is the contact resistance. In the present structure, the TLM geometry was used primarily to separate the channel-length-dependent resistance from length-independent contributions. Although pV3D3 is a dielectric material, its finite electrical conductivity allows a measurable resistance to be obtained between the metal electrodes. For samples exhibiting a linear resistance–length relationship, the slope therefore represents the effective length-dependent resistance term, whereas the y-intercept contains the resistance contribution that is independent of channel length, including the metal/pV3D3 contact contribution. Accordingly, *R_c_* is treated here as an effective contact resistance for comparative analysis rather than as an intrinsic metal/polymer contact resistivity. The total resistance was measured as a function of channel length, and the resistance–length relationship was compared among the reference, O_2_ plasma-treated, and thermally annealed samples. One sample was prepared for each processing condition, and multiple electrode structures were fabricated on each sample for electrical characterization. For C–V measurements, 29, 26, 24, and 21 individual electrode measurement records were obtained for the reference, O_2_ plasma-treated, 250 °C-annealed, and 300 °C-annealed samples, respectively. For TLM characterization, the corresponding numbers of resistance measurements were 82, 73, 76, and 95, including repeated measurements of certain electrode structures.

## 3. Results

### 3.1. FT-IR Analysis

**Figure 2:**
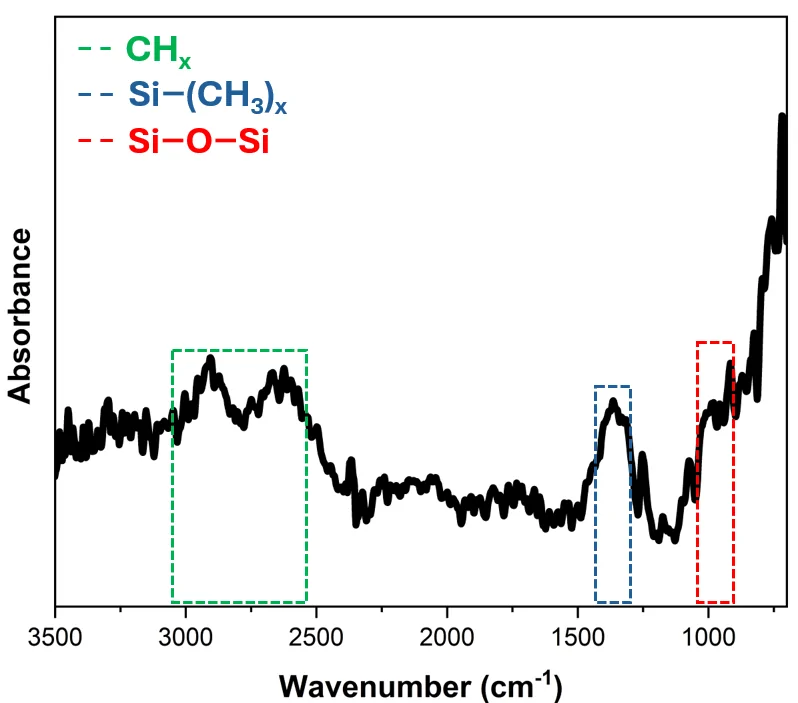
FT-IR spectrum of the as-deposited pV3D3 film.

Figure 2 shows the ATR-FTIR spectrum of the as-deposited pV3D3 film. The spectrum exhibited characteristic absorption bands in the C–H stretching region at approximately 3000–2800 cm^−1^, together with absorption features associated with Si–(CH_3_)_x_ at approximately 1400 cm^−1^ and Si–O–Si bonding in the range of approximately 1100–1000 cm^−1^ [10,17]. These characteristic absorption features confirm the chemical characteristics of the deposited pV3D3 film. ATR-FTIR measurements were performed only on the as-deposited pV3D3 film. Therefore, structural changes induced by O_2_ plasma treatment and thermal annealing could not be directly evaluated by FT-IR in this study. Accordingly, FT-IR is used here only to confirm the characteristic chemical structure of the as-deposited film.

### 3.2. Surface Morphology

**Figure 3:**
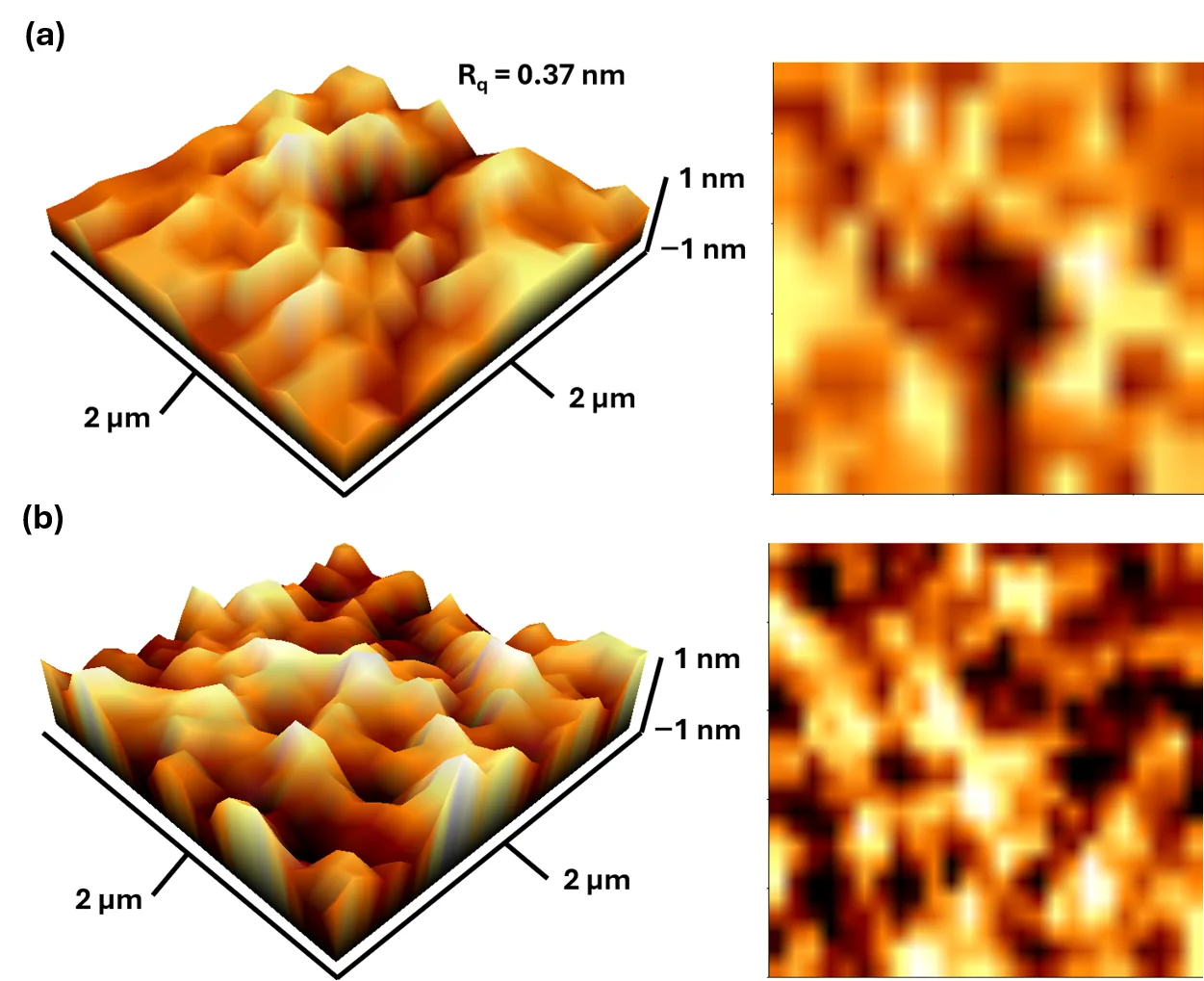
AFM images and RMS surface roughness of (**a**) reference and (**b**) O_2_ plasma-treated pV3D3 films.

Figure 3 shows the AFM images of the reference and O_2_ plasma-treated pV3D3 films. The reference film exhibited a smooth surface with a root-mean-square (RMS) roughness of 0.37 nm. After O_2_ plasma treatment, the RMS roughness was 0.48 nm, indicating only a minor change in surface roughness. Both films maintained smooth and uniform surface morphologies, consistent with the smooth surface characteristics previously reported for V3D3-derived polymer films [18], without noticeable surface damage.

These results indicate that the applied O_2_ plasma condition did not significantly alter the surface morphology of the pV3D3 film. Although the plasma treatment may modify the surface chemical state, as further examined by XPS, the nanoscale surface roughness was largely preserved. AFM measurements were not performed on the thermally annealed samples; therefore, no conclusion is drawn regarding annealing-induced changes in surface roughness.

### 3.3. XPS Analysis

**Figure 4:**
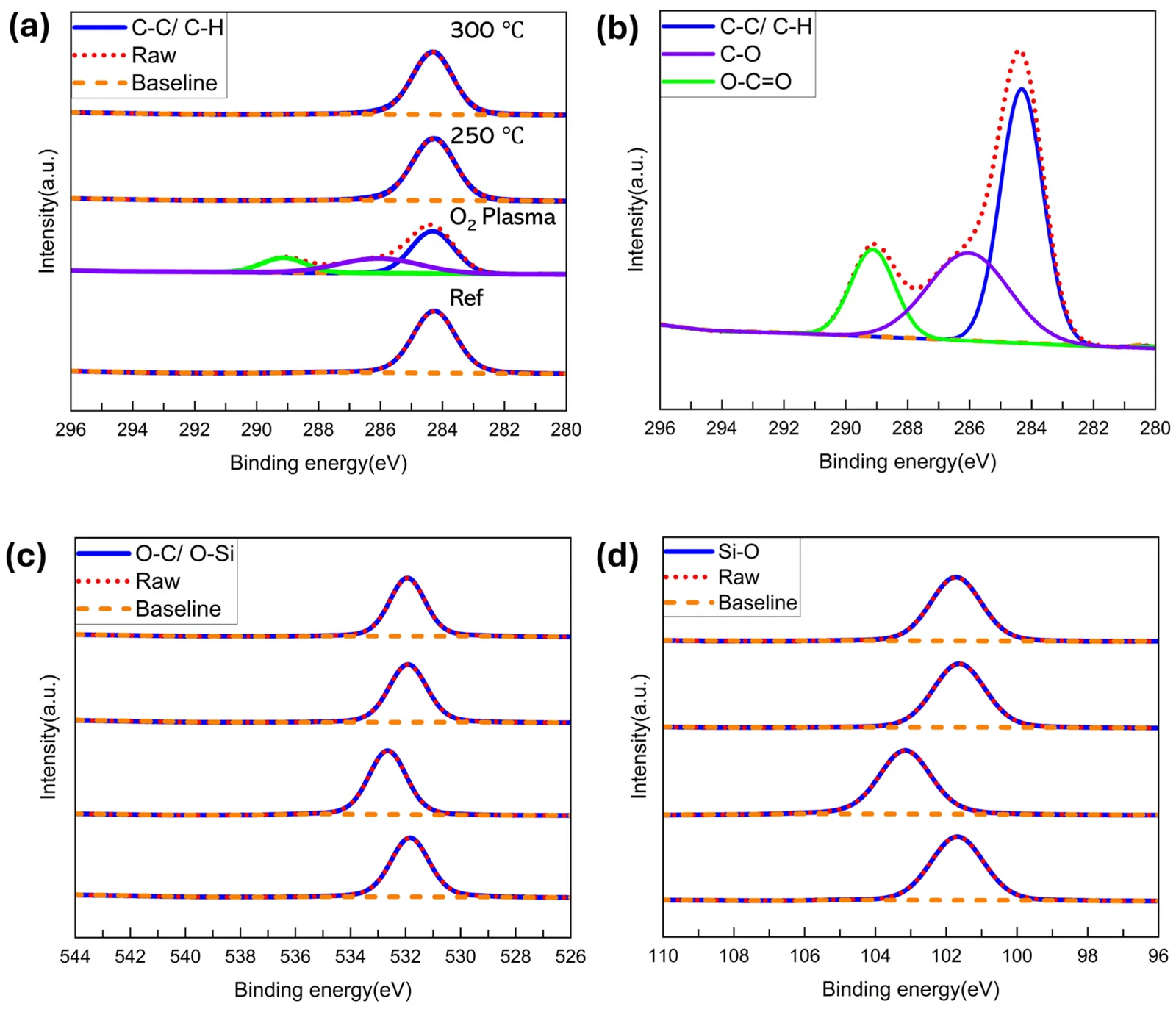
XPS spectra of pV3D3 films under different processing conditions: (**a**) C 1s spectra; (**b**) deconvoluted C 1s spectrum of the O_2_ plasma-treated pV3D3 film; (**c**) O 1s spectra; and (**d**) Si 2p spectra.

XPS analysis was performed to investigate changes in the surface chemical states of pV3D3 induced by O_2_ plasma treatment and thermal annealing. Figure 4a compares the C 1s spectra of the reference, O_2_ plasma-treated, and thermally annealed pV3D3 films. The reference and thermally annealed samples exhibited a dominant C 1s peak associated with C–C/C–H bonding, whereas the O_2_ plasma-treated sample showed additional contributions at higher binding energies, indicating a pronounced modification of the carbon bonding environment.

To further examine the chemical modification induced by O_2_ plasma treatment, the C 1s spectrum of the plasma-treated sample was deconvoluted, as shown in Figure 4b. The spectrum was resolved into C–C/C–H, C–O, and O–C=O components centered at 284.31, 285.8, and 289.13 eV, respectively, with corresponding relative peak areas of 45.33%, 37.75%, and 16.90%. The presence of the C–O and O–C=O components indicates an increased contribution of oxygen-containing carbon bonding states following O_2_ plasma treatment.

Figure 4c,d show the O 1s and Si 2p spectra, respectively. The O 1s spectra exhibited contributions associated with O–C/O–Si bonding environments under all processing conditions. Compared with the reference samples, the O_2_ plasma-treated sample exhibited a shift toward higher binding energies, with the O 1s peak shifting from 531.84 to 532.65 eV, corresponding to a binding-energy shift of 0.81 eV. A more pronounced shift was observed in the Si 2p spectrum of the O_2_ plasma-treated sample, with the peak shifting from 101.64 to 103.11 eV, corresponding to a binding-energy shift of 1.47 eV. In contrast, the samples annealed at 250 and 300 °C exhibited only minor changes in the O 1s and Si 2p peak positions relative to the reference sample, indicating no substantial binding-energy shift after thermal annealing. The higher-binding-energy shift of the Si 2p signal is consistent with a change toward a more oxidized Si-related chemical environment after O_2_ plasma treatment.

Taken together, the emergence of oxygen-containing C 1s components and the binding-energy changes observed in the O 1s and Si 2p spectra demonstrate that O_2_ plasma treatment induces a distinct modification of the near-surface chemical environment of pV3D3. In contrast, thermal annealing produced comparatively limited changes in the XPS spectra under the investigated conditions. Previous FTIR and XPS investigations of pV3D3 have discussed changes in Si-containing bonding environments associated with thermal curing and O_2_ plasma treatment, including possible changes among D-type (O_2_SiR_2_), T-type (O_3_SiR), and Q-type (O_4_Si) siloxane structures [19]. The higher-binding-energy shift observed in the Si 2p spectrum in the present study is consistent with a more oxidized Si-related chemical environment following O_2_ plasma treatment. However, because FT-IR measurements were not performed on the plasma-treated films, the formation and relative proportions of specific D, T, and Q units cannot be determined conclusively from the present XPS results alone.

### 3.4. Capacitance–Voltage Characteristics

**Figure 5:**
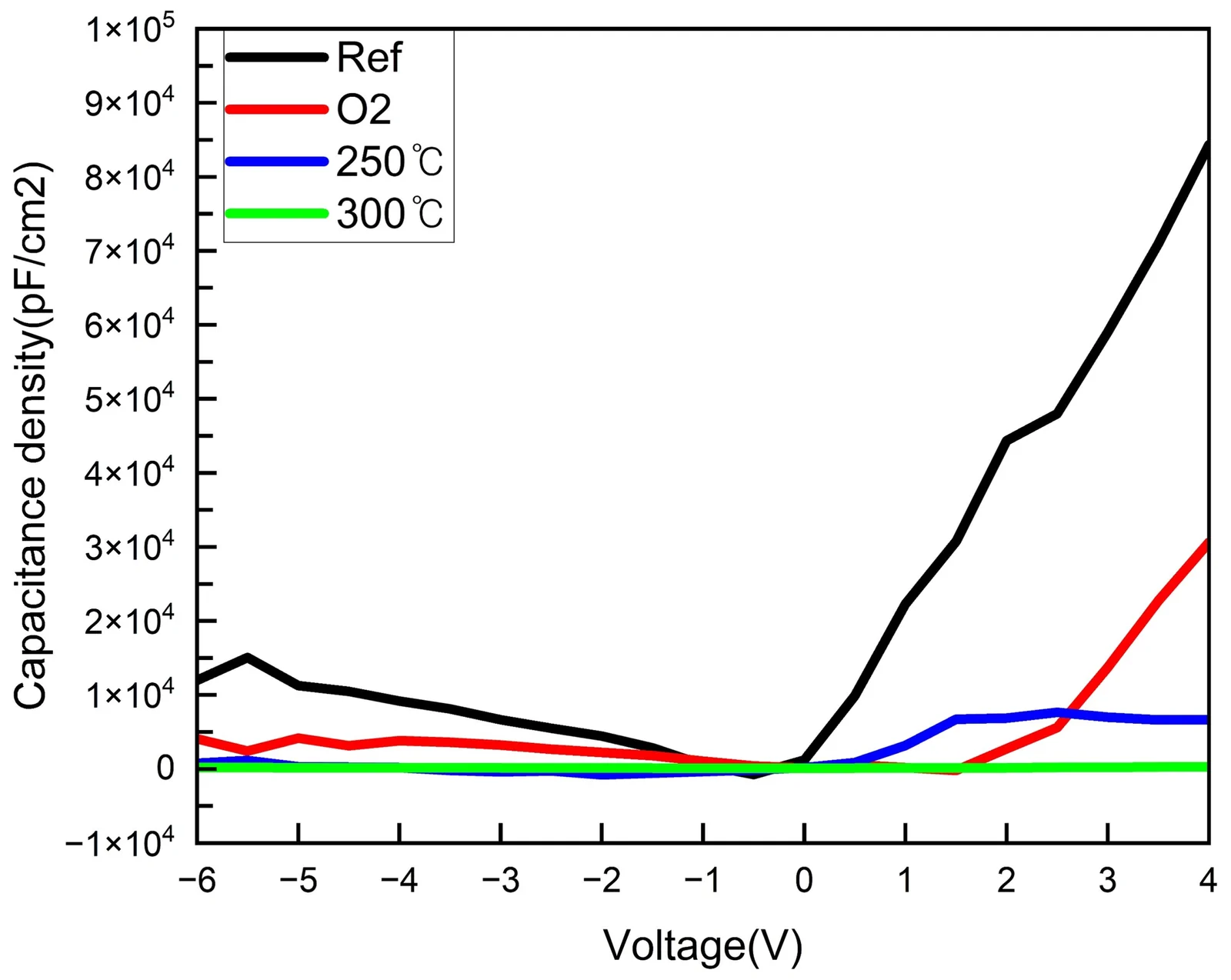
C–V characteristics of pV3D3 films under reference, O_2_ plasma-treated, and thermally annealed conditions.

Figure 5 shows the C–V characteristics of the pV3D3 films under different processing conditions. The measured capacitance was converted to capacitance per unit area for comparison among the samples. The reference sample exhibited a pronounced increase in capacitance density with increasing positive bias, whereas the processed samples showed condition-dependent changes in the capacitance response.

O_2_ plasma treatment reduced the capacitance density compared with the reference, while the sample annealed at 250 °C exhibited a further suppressed capacitance response. The most pronounced change was observed after annealing at 300 °C, for which the capacitance remained substantially lower over the investigated voltage range. These results indicate that thermal treatment, particularly at 300 °C, substantially modifies the effective electrical response of the pV3D3-based structure. However, the measured capacitance may also be influenced by leakage current, resistive contributions, and frequency-dependent effects. Since the C–V measurements were performed only at 10 kHz, these contributions cannot be independently distinguished from changes in the intrinsic dielectric properties of pV3D3. The slightly negative capacitance values observed in certain voltage regions may be associated with measurement-related effects, particularly resistive contributions, as suggested by the high dissipation-factor values in the raw measurement data. Because the original instrument correction settings were not retained, the small negative values observed in the high-dissipation regions are treated as measurement-related features and are not used to infer the intrinsic dielectric behavior of pV3D3.

### 3.5. Resistance Length Analysis Using TLM Structures

**Figure 6:**
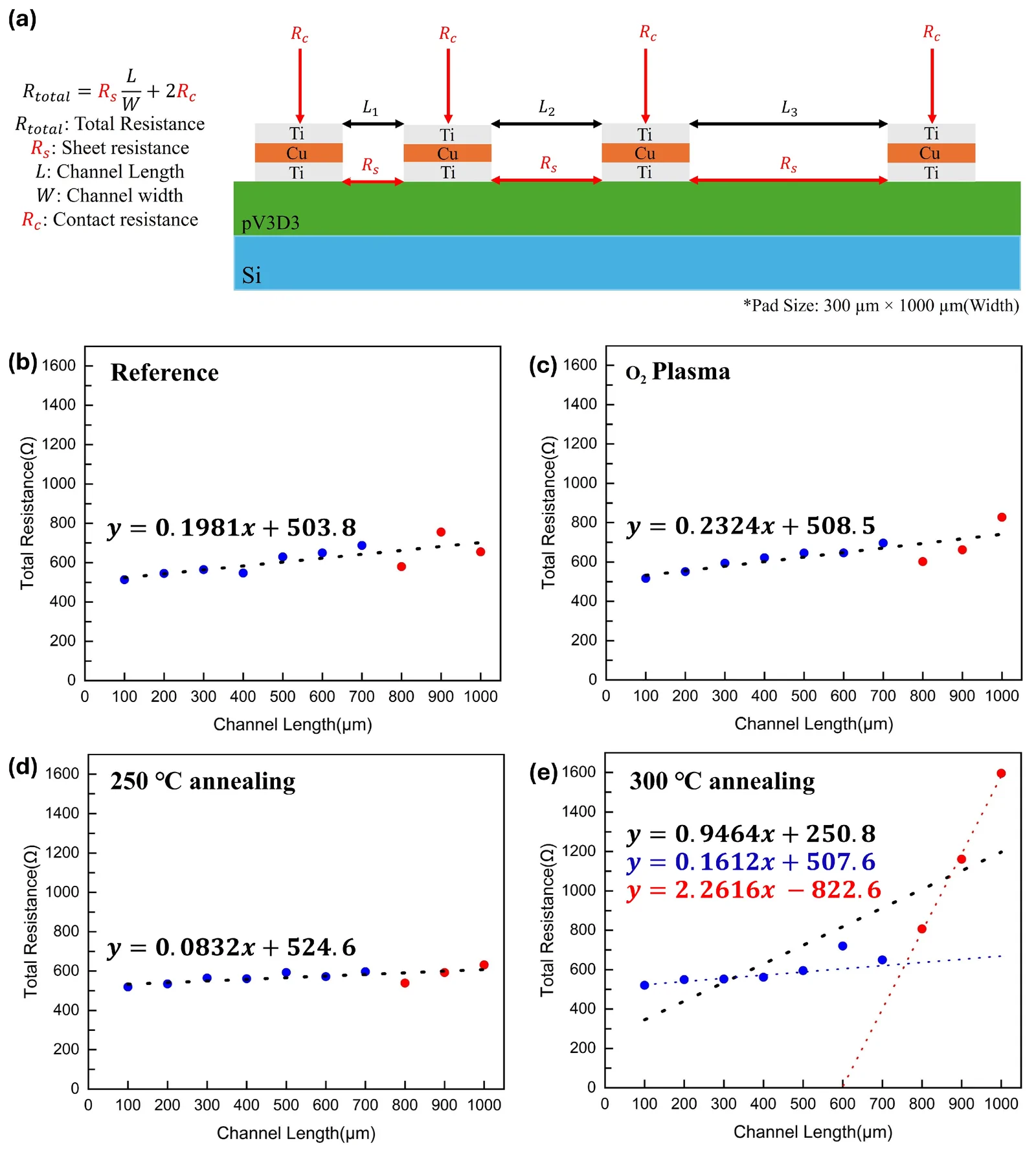
TLM structure and total resistance as a function of channel length for pV3D3 samples: (**a**) schematic of sheet and contact resistances; (**b**) reference, (**c**) O_2_ plasma, (**d**) 250 °C annealing, and (**e**) 300 °C annealing. Blue and red dots indicate channel lengths of 100–700 and 800–1000 µm, respectively. Black, blue, and red dotted lines represent the overall, short-channel, and long-channel linear fits, respectively. Red labels in (**a**) indicate resistance components, while colored equations in (**e**) correspond to the fitted lines.

Figure 6a illustrates the TLM structure and the resistance components used for the analysis, where *R_s_*, *R_c_*, *L*, and *W* represent the sheet resistance, contact resistance, channel length, and channel width, respectively. Figure 6b–e show the total resistance as a function of channel length for the reference, O_2_ plasma-treated, and thermally annealed pV3D3 samples. The reference and O_2_ plasma-treated samples exhibited similar linear resistance–length relationships, with fitted slopes of 0.1981 and 0.2324 Ω/µm, respectively. The sample annealed at 250 °C also showed a relatively weak dependence on channel length, with an overall fitted slope of 0.0832 Ω/µm. The y-intercepts obtained from the linear fits were 503.8, 508.5, and 524.6 Ω for the reference, O_2_ plasma-treated, and 250 °C-annealed samples, respectively. Based on the TLM relation, the corresponding intercept-derived effective contact resistances were approximately 251.9, 254.3, and 262.3 Ω, for the reference, O_2_ plasma-treated, and 250 °C-annealed samples, respectively. The relatively small variation among these values indicates that the length-independent resistance contribution remained comparable under these processing conditions [20,21].

In contrast, the sample annealed at 300 °C exhibited a distinct deviation from the approximately linear resistance–length behavior observed under the other processing conditions. The overall fit yielded a substantially increased slope of 0.9464 Ω/µm and a reduced y-intercept of 250.8 Ω. Furthermore, the resistance behavior exhibited a clear dependence on the channel-length regime. The shorter-channel region showed a slope of 0.1612 Ω/µm and a y-intercept of 507.6 Ω, whereas the longer-channel region exhibited a markedly increased slope of 2.2616 Ω/µm and a negative y-intercept of −822.6 Ω. These results demonstrate a clear deviation from a single linear resistance–length relationship after annealing at 300 °C, indicating that the conventional TLM assumption is no longer valid over the entire channel-length range. The separate fits in the shorter- and longer-channel regimes are therefore used only to describe the observed length dependence and are not interpreted as independent sheet or contact resistance values.

## 4. Discussion

O_2_ plasma treatment mainly affected the surface chemical state of pV3D3. The plasma-treated film exhibited C–O and O–C=O components in the C 1s spectrum, together with higher-binding-energy shifts in the O 1s and Si 2p spectra. These changes are consistent with an increased contribution of oxygen-containing bonding environments at the polymer surface. At the same time, the RMS roughness changed only from 0.37 to 0.48 nm, indicating that the plasma condition used here altered the surface chemistry without producing substantial nanoscale roughening. This behavior is consistent with previous reports showing that plasma treatment can modify the surface properties of pV3D3 while preserving a relatively smooth polymer surface [14,15].

Thermal annealing produced a different electrical response. The reference, O_2_-plasma-treated, and 250 °C-annealed samples retained an approximately linear resistance–length relationship, allowing the length-dependent resistance to be separated from the length-independent contribution represented by the intercept. The similar intercept values for these samples indicate that the effective contact-related resistance remained relatively unchanged under these conditions. In contrast, the 300 °C-annealed sample showed a pronounced departure from a single linear resistance–length relationship. The fitted intercept for this condition therefore cannot be interpreted as a conventional contact resistance. One possible origin of this behavior is a channel-length-dependent redistribution of the current path. Because pV3D3 is highly resistive but not an ideal insulator, thermal modification of the polymer or the pV3D3/Si interface may alter the effective conduction path as the electrode spacing increases. Field-dependent transport may also contribute because the electric field at a fixed applied voltage decreases with increasing channel length. The separate short- and long-channel fits are therefore used only to describe the observed deviation from linear TLM behavior rather than to extract independent material parameters.

The relatively small change in the surface-sensitive XPS spectra, despite the much larger electrical response after thermal annealing, suggests that the origin of the electrical modification is not limited to the near-surface chemical state. Possible contributions include structural rearrangement within the 50-nm-thick polymer layer, changes at the pV3D3/Si interface, and process-induced defect states [22]. Thermally driven rearrangement and ring opening of cyclosiloxane-based polymer structures have been reported previously [16]. However, post-annealing FT-IR, film-thickness, and depth-resolved measurements were not performed in this study, so the microscopic origin of the observed electrical changes cannot be identified conclusively.

The comparison nevertheless shows a clear difference between the two processing routes investigated here. O_2_ plasma treatment predominantly changed the near-surface chemical environment while maintaining a smooth surface, whereas annealing at 300 °C produced the largest change in the electrical response. The agreement between the C–V and resistance–length measurements indicates that thermal budget is an important consideration when pV3D3 is incorporated into semiconductor process flows.

## 5. Conclusions

This study comparatively evaluated the effects of O_2_ plasma treatment and thermal annealing on 50 nm thick iCVD pV3D3 dielectric films. O_2_ plasma treatment produced distinct oxygen-containing surface chemical states while maintaining sub-nanometer surface roughness. In contrast, thermal annealing, particularly at 300 °C for 3 h, caused pronounced changes in the C–V response and resistance–length characteristics despite relatively limited changes in the surface-sensitive XPS spectra. This discrepancy suggests that thermally induced electrical modification may involve bulk or interfacial effects that are not fully captured by surface characterization alone. These results demonstrate that the thermal budget should be carefully considered when integrating pV3D3 into semiconductor process flows. The 300 °C condition identified here should be regarded as a process-specific condition producing significant electrical modification rather than as a universal thermal stability limit of pV3D3.

## Data Availability

The data presented in this study are available on request from the corresponding author due to institutional data management limitations and the large volume of raw measurement files.
